# Efficient data collection for establishing practical identifiability via active learning

**DOI:** 10.1016/j.csbj.2025.10.058

**Published:** 2025-10-29

**Authors:** Xiaolu Liu, Linda Wanika, Michael J. Chappell, Juergen Branke

**Affiliations:** a Mathematics Institute, University of Warwick, Coventry CV4 7AL, United Kingdom; b School of Engineering, University of Warwick, Coventry CV4 7AL, United Kingdom; c Warwick Business School, University of Warwick, Coventry CV4 7AL, United Kingdom

**Keywords:** Practical identifiability, Active learning, Bayesian experimental design, Profile likelihood, Parameter estimation, Systems biology

## Abstract

Practical identifiability analysis (PIA) plays a crucial role in model development by assessing whether the available data are sufficient to yield reliable parameter estimates. In bioengineering applications, identifying the minimal experimental design that ensures parameter identifiability is essential in order to reduce cost, time, and resource consumption. In this paper, we introduce E-ALPIPE, a sequential active learning algorithm that recommends new data collection points most likely to establish practical identifiability given the current data, mathematical model and noise assumptions. We empirically evaluate E-ALPIPE against both a benchmark algorithm from the literature and random sampling over three synthetic experiments. Our results show that E-ALPIPE substantially reduces the number of observations required to achieve practical identifiability, while producing comparable or narrower confidence intervals and more accurate point estimates of system dynamics.

## Highlights

• Active learning for practical identifiability analysis.
• Achieves practical identifiability with less data while improving parameter estimates.
• For linear or nonlinear systems, and systems with multiple outputs.
• Uses binary search to efficiently determine profile likelihood confidence intervals.

## Graphical Abstract

**Figure:**
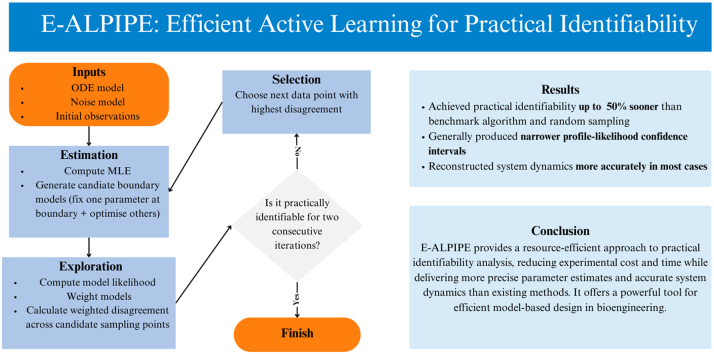

## 1 Introduction

Models of bioengineering systems have enabled significant advances in biological understanding and medical research by providing quantitative frameworks to describe complex biological systems [1], [2], [3]. In recent years, hybrid approaches that combine mechanistic and data-driven modelling have gained increasing popularity [4]. This combination allows parameters in mechanistic models to be estimated from experimental data, while simultaneously validating whether model simulations accurately reproduce the underlying biological phenomena.

Mathematical models in bioengineering typically consist of ordinary differential equations to describe the dynamics of a biological process over time. For such models to be reliable, their parameters must be identifiable — that is, recoverable uniquely (or locally) and with acceptable precision from experimental data. Identifiability analysis has two components: structural identifiability analysis (SIA) and practical identifiability analysis (PIA) [5]. SIA is a pre-requisite for PIA and assesses the identifiability of parameters based on the model structure alone and on the assumption of continuous, noise-free observations, whereas PIA extends this concept to real-world scenarios involving noisy and finite datasets.

Different strands of PIA research are based on different theoretical frameworks, including considerations of profile likelihoods, the Fisher information matrix (FIM), Markov Chain Monte Carlo (MCMC) and the Bayesian Information Criterion [6]. The FIM PIA is mainly applicable to linear systems and thus has relatively limited use for biological models [7]. Bayesian MCMC methods are more general but can often prove to be very expensive computationally and may also suffer from poor chain mixing or strong dependence on prior assumptions [7]. The approach based on the Bayesian Information Criterion [8], [9] can be seen as sitting between SIA and PIA as it does not use real data but does take into account noise and finite sample sizes.

Our work builds on the profile likelihood approach proposed in [5] and discussed in more detail in the Background section. In this approach, the parameter of interest is varied over a region around its estimate, and the likelihood is evaluated when all the remaining parameters have been re-optimised.

Establishing identifiability often depends critically on how experiments are designed. In practice, experiments usually have to be repeated multiple times to generate what is deemed “sufficient” data for model development [5]. Although this strategy may eventually yield adequate parameter estimates, it can be time-consuming, costly, and resource-intensive [10]. Based on the ideas of active learning, modern optimal experimental design (OED) provides an attractive alternative: by iteratively selecting the most informative next experiment, OED enables more accurate parameter estimation with fewer experiments, thereby reducing cost and experimental effort [10], [11], [12]. Among OED approaches, Bayesian Optimal Experimental Design (BOED) optimises the expected information gain over the posterior, which typically involves repeated posterior sampling or mutual-information approximations and can be computationally prohibitive [10] for non-linear dynamic models such as those considered in this paper. We therefore focus on a profile-likelihood-based approach and develop an algorithm that directly targets practical identifiability.

A few previous studies have proposed the use of profile likelihoods to guide experimental design. In [13] the authors show, based on a nonlinear signal pathway model, how to use PIA to identify additional model states that should be observed in order to render the model practically identifiable. The basic concept is to examine the variability of the model prediction at various model states, and then add the model state to the set of observed experimental outputs that exhibit the largest variability. Intuitively, such observations are most effective in constraining the parameter space compatible with experimental evaluations, as the new data will necessarily disagree with many parameter settings. However, augmenting the model states to be observed with additional states typically requires modifying the experimental setup and is thus often not possible if the desired state cannot be practically measured. Other studies [14], [15] utilise the same foundational profile likelihood concepts as [13], but rather than augmenting the observations with additional model states, they consider adding observations of existing model states but at different time points, something that in practice is often easier to perform. These authors also propose an explicit formula for the variability of model predictions which takes into account the noise present, essentially favouring times that have a high signal-to-noise ratio [14]. Their setting is closest to the one that we present in this paper, and thus we utilise their method as the benchmark in our empirical evaluations. Finally, [16] assumes access to a complete experimental dataset to identify minimally sufficient experiments. While insightful, such analysis of an a *posteriori* nature is generally not feasible as a general experimental design strategy in practice.

In this work, we propose the Efficient Active Learning Practical Identifiability Parameter Estimation algorithm (E-ALPIPE), which introduces a novel active learning criterion for optimal experimental design, directly targeted at improving practical identifiability as defined by the profile likelihood. Unlike previous profile likelihood-based methods that rely on simple disagreement heuristics, E-ALPIPE adaptively selects experiments based on likelihood-weighted disagreement among candidate models. We empirically demonstrate that this new criterion is computationally efficient, requires fewer experiments (time points for data collection) to establish practical identifiability and achieves comparable or better parameter accuracy compared to the previous state-of-the-art.

The structure of this paper is as follows: Section 2 reviews the theoretical foundations. Section 3 details our E-ALPIPE algorithm, while the benchmark algorithm is described in Section 4. Empirical results are presented in Section 5. Finally, Section 6 discusses the current limitations of the approach, contributions, and directions for future research.

## 2 Background

This section provides a brief introduction to the principles of uncertainty sampling in active learning as well as practical identifiability analysis using the profile likelihood approach.

### 2.1 Uncertainty sampling within active learning

Active Learning is an influential paradigm in both supervised and semi-supervised machine learning, and is especially effective in optimising model performance under a restricted budget [17]. It is particularly beneficial in scenarios with high costs for acquiring labelled data [18]. Its goal is to maximise model performance under a limited labelling budget by selecting only the most informative unlabelled instances for annotation. The criterion for what constitutes informative data varies across algorithms, but universally aims to enhance model accuracy with fewer labelled instances, thereby minimising the associated costs [19]. Recent work has also demonstrated the utility of active learning in experimental biology, for example in optimising experimental design in biotechnology using active learning strategies [20].

Among the various data selection criteria, Uncertainty Sampling is the most widely studied: at each step, the algorithm queries the point whose predicted label is least certain under the model, then updates the model with that newly labelled data [17].

Common measures of prediction uncertainty include variance, least confidence and entropy [17]. However, the detailed interpretation of uncertainty varies depending on the specific requirements of different research approaches [21]. In Section 3 we propose a metric that can take into account varying noise levels and multiple outputs.

### 2.2 Structural identifiability analysis

Structural identifiability analysis is a prerequisite for a model to be considered practically identifiable [22]. SIA assesses, based on the structure of a given model system (the model’s inputs, outputs, and the unknown parameters), whether a unique, finite, or infinite set of values for the unknown parameters can reproduce the model’s output(s). Several methods exist for SIA, including the Laplace transform approach (for linear systems), Taylor series expansions, and input–output relationships derived using Lie derivatives [7], [23], the latter being applicable to both linear and nonlinear system models. A structurally unidentifiable parameter would indicate that infinitely many values for that unidentifiable parameter can yield exactly the same output even under the conditions of noise free and “perfect” data [24]. Structural unidentifiability is typically addressed through model reformulation, model reparameterisation or experimental redesign (e.g., potentially requiring the observation of certain additional model states).

### 2.3 Practical identifiability analysis – profile likelihood approach

Practical identifiability analysis determines whether model parameters can be reliably estimated from the available experimental data. Various methods exist for assessing practical identifiability, including the Markov Chain Monte Carlo (MCMC), the Fisher information matrix, and profile likelihood analysis [6]. Comparison studies show that profile likelihood can provide similar conclusions to MCMC approaches, but typically incurs lower computational cost because it avoids posterior sampling and potential mixing issues [25]. Given its efficiency and widespread use in biological and bioengineering system models [5], [7], we have chosen to adopt the profile likelihood approach in this paper.

**Fig. 1:**
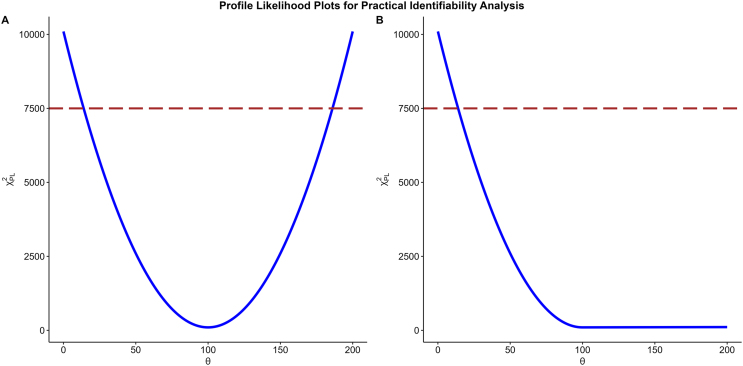
Profile likelihood plots for parameter of interest (θ) (A) a practically identifiable parameter and (B) a practically unidentifiable parameter. Blue curve is the profile likelihood and brown dashed line represents the 95 % profile likelihood-based confidence interval threshold. (For interpretation of the references to colour in this figure legend, the reader is referred to the web version of this article.)

This approach systematically examines how the likelihood function changes as one parameter varies while optimising all other parameters to best fit the data. By analysing the shape and width of the resulting profile likelihood curves, one can evaluate the precision and uniqueness of the parameter estimates obtained. In [5], the authors demonstrate how the shape of the profile likelihood can be used to assess parameter identifiability: a profile that is flat on at least one side of the parameter estimate indicates practical non-identifiability, whereas a sharply curved peak or trough suggests practical identifiability (see Fig. 1). These qualitative differences provide a visual diagnostic for practical identifiability, while the corresponding likelihood-ratio test quantifies the associated statistical confidence. Building on this concept, later studies have used prediction disagreement derived from profile likelihoods to suggest additional measurement time points or hidden states that could improve parameter identifiability [13], [14], [15].

The likelihood of a parameter can be interpreted as the probability of generating the observed output given that parameter, i.e.,

(1) L(θ∣y)=P(y∣θ),

where θ refers to a parameter vector, y is the vector of observed outputs, L is the likelihood, and P is the associated probability.

In many hybrid modelling and data-driven approaches, the likelihood serves as a basis for parameter estimation. Modifications in parameter estimates can change the overall likelihood, which is the basis of the profile likelihood approach. Briefly, each unknown parameter has a Maximum Likelihood Estimator (MLE) and an associated likelihood value.

To compute the profile likelihood for a parameter $\theta_{i}$ of interest, we discretise $\theta_{i}$ while re-optimising the remaining nuisance parameters $\theta_{j\neq i}$ to maximise the likelihood for each fixed value of $\theta_{i}$. If $\theta_{i}$ is practically identifiable, we expect a significant change in the likelihood when $\theta_{i}$ is varied. Eq. (2) describes how the 95 % profile likelihood confidence interval is computed, where $\theta_{\text{MLE}}$ is the MLE of parameter $\theta_{i}$, $nLL(\theta_{\text{MLE}})$ is the negative log likelihood of the MLE, and $\chi^{2}(0.95,1)$ is the 95th percentile of the chi-squared distribution with one degree of freedom, as the calculated confidence intervals are point-wise based for each parameter [5]:

(2) $$\text{threshold}=nLL(\theta_{\text{MLE}})+\frac{\chi^{2}(0.95,1)}{2}.$$

If the profile likelihood values exceed the threshold on both sides of the MLE, we conclude that the parameter is practically identifiable [5] (see Fig. 1). The values of $\theta_{i}$ at which the likelihood crosses the threshold on each side of the MLE define the 95 % confidence interval. For practically unidentifiable parameters one or both sides of the likelihood curve will not cross this threshold.

Since the discretised values of $\theta_{i}$ play an important role in the generated likelihood values and identifiability conclusions, the discretisation strategies are important. Standard tools use fixed or adaptive grids (e.g. 100 points per side), which can be costly [26], [27], [28]. To address this, in Section 3.4 we introduce a targeted bisection-based boundary search that reduces computational overheads while preserving accuracy in practical identifiability assessments.

For a more in-depth introduction to practical identifiability analysis, we refer to [5], [22].

## 3 The efficient active learning practical identifiability parameter estimation (E-ALPIPE) algorithm

Like other active learning based approaches to experimental design, our approach works with a set of candidate models and examines the variability or disagreement between their predictions. We first discuss these two components – the selection of candidate models and the disagreement metric – before we explain the algorithm in detail. We also introduce an efficient search strategy to accelerate practical identifiability assessment.

The overall procedure iteratively evaluates and refines model identifiability through adaptive experimental design. At each iteration, if the parameters remain unidentifiable, the algorithm selects additional sampling time points that maximise a likelihood-weighted disagreement among candidate models. This process is repeated until all parameters are practically identifiable or a predefined iteration limit is reached.

### 3.1 Candidate models

E-ALPIPE considers a set of candidate models in each iteration which are derived from a simplified profile likelihood analysis.

Consider a candidate model parameterised by a vector θ, and existing observations $D=\{(y_{1},x_{1}),(y_{2},x_{2}),\ldots ,(y_{n},x_{n})\}$, where $x_{j}$ denotes the experimental condition (e.g., the sampling time or input setting) associated with noisy observation $y_{j}$. Let the noise-free model output at condition x given parameters θ be denoted by f(x∣θ). Under a Gaussian noise model with standard deviation σ and assuming the noise is independent across observations, the corresponding likelihood function is given by

(3) $$L(\theta |D,\sigma )=\prod_{j=1}^{n}\frac{1}{\sigma }\;\phi \;\left(\frac{y_{j}-f(x_{j}|\theta )}{\sigma }\right),$$

where ϕ denotes the standard normal PDF.

The likelihood formulation can be directly generalised to alternative noise models. For example, in the experiments below we use a truncated normal noise model, where Eq. (3) is replaced by Eq. (A.1) (see Appendix A for details).

The first candidate model considered by E-ALPIPE is the maximum likelihood model, obtained by maximising Eq. (3) over θ. The remaining candidate models are the boundary models from profile likelihood analysis, obtained by fixing one parameter at its lower or upper boundary value and maximising the likelihood over all the other parameters. In total, this generates m=2N+1 candidate models for a system with N parameters.

### 3.2 Disagreement among candidate models

As discussed above, the disagreement among possible candidate models is a good indicator of where a sample may be informative. Our m candidate models $f(x|\theta_{1}),\ldots ,f(x|\theta_{m})$, are each defined by a distinct parameter vector $\theta_{i}$. However, not all candidate models are equally likely, and thus we weight their influence based on likelihood, which quantifies how well a model explains observed data.

By normalising the likelihood values across all candidates, we obtain relative weights

(4) $$w_{i}=\frac{L(\theta_{i}|D,\sigma )}{\sum_{k=1}^{m}L(\theta_{k}|D,\sigma )}.$$

These weights are updated at each iteration and guide the selection of the next experimental design. With these weights, we can compute, for each candidate location x, a weighted disagreement between the boundary models and the MLE model as:

(5) $$\text{Disagreement}(x)=\sum_{i=1}^{m}w_{i}{\left(f(x|\theta_{i})-f(x|\theta_{MLE})\right)}^{2}.$$

To account for differences in measurement uncertainty across observations, we follow the general principle of the signal-to-noise ratio (SNR) to normalise the information gain. Specifically, at each iteration, we first compute the most likely system trajectory using the current MLE estimate. For each observable, we then estimate the corresponding noise variance based on the current MLE and the known noise model (see Appendix A for details). The weighted disagreement is then normalised by dividing it by the predicted noise variance at each sampling location. Finally, for each candidate location, the normalised disagreements across M output dimensions are aggregated to obtain an overall acquisition score, defined by:

(6) $$\text{Scaled Disagreement}(x)=\sum_{j=1}^{M}\left(\frac{1}{{\hat{\sigma }}_{j}^{2}}\sum_{i=1}^{m}w_{i}{\left(f_{j}(x|\theta_{i})-f_{j}(x|\theta_{MLE})\right)}^{2}\right).$$

In summary, this criterion prioritises sampling locations where candidate models exhibit strong, weighted disagreement that cannot be explained by measurement noise, as such locations are expected to yield the highest information gain.

Algorithm 1 **Efficient Active Learning for Practical Identifiability Parameter Estimation**

### 3.3 Algorithm

Given the ODE and noise models, the algorithm begins with the initialisation, and then alternates between two phases:

1. *The estimation phase*: Identifies the Maximum Likelihood Estimate (MLE) model and the boundary models as explained in Section 3.1.
2. *The exploration phase*: Estimates the noise level at each possible sampling location and determines the next data collection point by selecting the point with the highest scaled disagreement among the predicted outputs from all the models generated during the estimation phase.

We iterate until practical identifiability is established or a maximum number of iterations has been reached. The algorithm is summarised in Algorithm 1, as follows:

#### Initialisation (Line 1–2)

We start with a set of random observations, noise model and the analytical form of the function.

#### Estimation (Lines 3–10)

With existing observations, we calculate an approximate MLE, and construct a set of boundary models. Each boundary model is found by fixing one parameter at its corresponding boundary value, and maximising the likelihood by optimising the other parameters using the commonly employed local optimisation algorithm l-BFGS with multi-start [29].

#### Decision on practical identifiability (Lines 11–17)

From the identified MLE parameters, we can construct the threshold for deciding practical identifiability based on Eq. (2). If all parameters are practically identifiable, and the last iteration was practically identifiable as well, we search for its profile likelihood confidence interval using a bisection search detailed in Section 3.4.

#### Searching for the next experiment (Lines 18–25)

We calculate and normalise the likelihood of all candidate models, obtaining a relative probability of each model being the true underlying model (see Section 3.2). Based on these weights, we compute the scaled disagreement score for each location and select the one with the highest scaled disagreement.

#### Data collection (Line 26)

We collect new data at the selected location (for all observed outputs) and add these to the dataset to inform the subsequent iterations.

#### Termination (Lines 16 and 27)

We iterate until practical identifiability has been established for two consecutive iterations to ensure stability of the results, or until we reach the maximum number of iterations (in the event that practical identifiability has not been attained).

### 3.4 Boundary search for profile likelihood confidence interval

Common practical identifiability analysis tools for bioengineering models are the data2dynamics modelling environment in MATLAB [26], the LikelihoodProfiler package in JULIA [28], and the pyPESTO package in PYTHON [30]. All of these tools and packages use the profile likelihood approach. In data2dynamics, for example, default profile likelihood computation explores up to 100 discretised values on either side of the MLE [26]. However, in some instances the default value of 100 steps is not sufficient to accurately identify the profile likelihood confidence intervals. To enhance the speed and accuracy of computing the profile likelihood confidence intervals, and assuming the profile likelihood is unimodal, we propose using bisection search [31]. This unimodality assumption is common in practical identifiability analysis of well-constrained biological models and ensures that the likelihood crosses the confidence threshold at most once on each side of the MLE. In cases where profiles are strongly multimodal, the bisection step can return wider confidence intervals; in that case one can use grid/multi-interval search around the MLE and boundary regions to ensure that threshold crossings are not missed. The algorithm works as follows:

• **Initialise:** With the MLE for the given data, we calculate the threshold for the practical identifiability check. Eq. (2) shows that the threshold value is only dependent on the confidence level and the MLE, and therefore this threshold can be shared for all parameter settings for which we are checking practical identifiability. We define the initial boundaries to be the user-defined parameter boundaries, and the error tolerance to be a user-defined value $\epsilon_{i}$ for each parameter.
• **Practical identifiability check:** For each parameter, we calculate the profile likelihood $pl_{min}$ and $pl_{max}$ at its boundaries. If either $pl_{min}\leq threshold$ or $pl_{max}\leq threshold$, we conclude that this parameter is not (yet) practically identifiable.
• **Profile likelihood confidence interval calculation:** Two bisection searches are performed: one between the parameter’s lower bound and the MLE, and the other between the MLE and the upper bound. These searches iteratively refine the interval and return the estimated lower and upper bounds that define the confidence interval for the parameter under evaluation [32]. If the profile likelihood is continuous, the bisection search will locate a boundary where the likelihood crosses the confidence threshold. In the case of a monotonic profile likelihood, the algorithm guarantees the smallest interval satisfying the confidence criterion. The bisection search terminates once the interval width falls below the tolerance $\epsilon_{i}$ specified in initialisation stage.

In practice, we found that it takes only 2–13 iterations to find the intersection point on each side of the parameter estimate, substantially fewer than traditional grid-based methods.

## 4 Benchmark algorithm

The benchmark algorithm that we compare to was proposed in [13] and, to the best of our knowledge, is the only existing method that explicitly addresses experimental design for improving practical identifiability using profile likelihood. Specifically, it determines model outputs from a number of models along their profile likelihood paths, i.e., when each parameter is fixed at a range of values with the other parameters re-optimised. Then, it suggests taking an additional measurement where the predicted outputs of these models vary the most, as they are more likely to reduce uncertainty and tighten the confidence intervals associated with practically non-identifiable parameters [13]. The original work by Raue et al. [13] focused on adding new observable states, whereas the experimental design in subsequent studies and in our work aims to identify the most informative new sampling time points of already observed states.

Steiert et al. [14] and Jeong et al. [15] demonstrate that the approach is practical in real world experimental settings. In their framework, for each candidate time point, the model is simulated across all acceptable parameter settings derived from the profile likelihood trajectories, and the experiment yielding the widest predicted output range divided by the noise standard deviation is selected. We follow the same overall principle by evaluating the disagreement between candidate model trajectories generated along the profile likelihood paths. Since the previous studies do not specify how the parameter space was discretised when constructing these trajectories, here, we adopted a simple and reproducible discretisation scheme: we pick 10 evenly spaced values for each parameter across its plausible range. In each iteration, if a parameter is deemed practically identifiable, we only consider the points within the estimated profile likelihood confidence interval. If no point falls within this interval, then we include the midpoint of the confidence interval to ensure coverage. For each selected value, we compute the corresponding constrained optimum using maximum likelihood estimation, generating a collection of at most 10×N profile likelihood models across the N parameters.

To select the next experimental measurement time point, the algorithm evaluates the variability among the generated model trajectories by computing, for each candidate time point, the variance of the predicted values. The candidate time point with the highest variance is selected. In the original studies, this procedure was applied to single-output models, where the variance is a scalar quantity.

To adapt the benchmark algorithm to settings with multiple outputs, we replace the variance with the generalised variance which corresponds to the determinant of the covariance matrix. Furthermore, since the measurement noise in our system depends on the magnitude of the observations, we use a scaled variant akin to the signal-to-noise ratio and following the same principle as the metric proposed in [14], by dividing the disagreement metric by the product of the estimated noise variances.

The estimation of the noise variance for truncated normal distribution is detailed in Appendix A. For each candidate sampling location x, we compute the relative variance R(x) by dividing the predicted output generalised variance by the estimated noise variance. Using two-dimensional outputs as an example, let $f(x|\theta )={\left[f_{1}(x|\theta ),f_{2}(x|\theta )\right]}^{T}$represent the matrix of predicted model outputs for the sampled parameter vector θ, and $\sigma_{1}$ and $\sigma_{2}$ denote the corresponding estimated noise variances. The mathematical expression for our relative variance R(x) is then defined as follows:

(7) $$R(x)=\frac{1}{\sigma_{1}^{2}\sigma_{2}^{2}}\det\left[\begin{array}{cc} {\text{Var}}_{\theta }(f_{1}(x,\theta )) & {\text{Cov}}_{\theta }(f_{1}(x,\theta ),f_{2}(x,\theta ))\\ {\text{Cov}}_{\theta }(f_{1}(x,\theta ),f_{2}(x,\theta )) & {\text{Var}}_{\theta }(f_{2}(x,\theta )) \end{array}\right].$$

This metric allows us to jointly consider multiple outputs with different levels of noise when making a data collection decision. Throughout the rest of the paper, we refer to the benchmark algorithm which has generalised variance replaced by this relative–variance criterion as the *Scaled Benchmark*.

## 5 Empirical results

In this section, we demonstrate the advantages of E-ALPIPE over the Benchmark, Scaled Benchmark algorithms and Random Sampling based on examples with one and two outputs. We first introduce the mathematical models used in this paper, define the noise distributions, and explain how we customise them for our research.

The key difference between the examined methods lies in the data collection process: the E-ALPIPE, Benchmark and Scaled Benchmark algorithms all actively select data, whereas the random sampling method collects data at random time points from the candidate pool of those available for each experiment. Compared to the Benchmark and Scaled Benchmark methods proposed in [13], E-ALPIPE differs in both its acquisition criterion and the set of models used to evaluate the information gain. Specifically, E-ALPIPE selects the next sampling point as the *argmax* of the weighted disagreement among the boundary models and the MLE model (Eq. (6)), whereas the Benchmark and Scaled Benchmark methods maximise the (scaled) prediction variance. Moreover, E-ALPIPE computes this disagreement using only 2N+1 boundary-based parameter settings (including the MLE model), while the Benchmark and Scaled Benchmark methods evaluate 10N uniformly spaced parameter values. This difference substantially reduces the number of optimisations and thus computational time required by E-ALPIPE.

The three performance metrics used here are as follows:

1. **Success rate and speed of determining practical identifiability:** For the same number of collected data points, we compare the percentage of replications that achieve practical identifiability. If a new data point provides information that conflicts significantly with the existing dataset, e.g., if the new observation is an outlier, this can introduce new uncertainty about the parameter values. Therefore, we consider an iteration to be reliably identifiable only if it is identifiable across two consecutive iterations. For each replication, we record the iteration at which the model first becomes reliably practically identifiable, defined as the second of two consecutive iterations that both satisfy the identifiability criterion. Reaching identifiability quickly is the motivation for our approach and thus this is our key performance criterion.
2. **Width of the profile likelihood confidence interval:** We analyse the evolution of the profile likelihood confidence interval widths across the first 20 iterations and report the results as the mean and standard error over all trials. Statistical comparisons are conducted at the 20th iteration.
3. **Point estimate quality:** We evaluate the quality of the MLE point estimates by plotting the mean and standard error of the integrated absolute difference between the predicted and true function responses over iterations 1–20. Statistical comparisons are conducted using the results at the 20th iteration. The specifics of this calculation are detailed later in Section 5.4.

In Experiment 1 below, all the results are averaged across **100** replications, while the number of replications is **50** in Experiments 2 and 3 due to the higher computational complexity of those two experiments. We terminate a run either when we reach reliable practical identifiability or when we have collected 50 data points (the default maximum for evaluating all algorithms). Each experiment commences with one observation at the same location which can be defined by the user. In this paper, the first observation for Experiment 1 is at t=3, and t=10 hours for Experiments 2 and 3. Then, iteratively additional data points are collected using each method. The practical identifiability and profile likelihood assessments are generated only from the second data point onwards. Finally, to assess statistical significance, we use the Wilcoxon signed-rank test, which does not assume normality and is therefore more appropriate for our output distributions.

### 5.1 Models used in the experiments

We use three example models for the experiments in this paper. The first model is a sum of exponentials, which was used in [15]. The other two models are based on a nonlinear bioreactor, which was adapted from the microbial growth model described in [33]. We consider two versions of this bioreactor model: one with a single output, and one with two outputs.

#### 5.1.1 Sum of two exponentials model

The first model considers the sum of two exponentially decaying components, with decay rates $\beta_{1}$, $\beta_{2}$ in units of ${\text{hr}}^{-1}$. Only their combined total is observed at discrete time points, while both components are assumed to start at 1 when t=0 hours. The system can be formulated as a set of ODEs (Eq. (8)) which has an analytical solution (Eq. (9)), as previously described in [15]:

(8) $$\left\{ \begin{array}{ll} \frac{dx_{1}}{dt} & \;=-\beta_{1}x_{1},\\ \frac{dx_{2}}{dt} & \;=-\beta_{2}x_{2},\\ y(t) & \;=x_{1}(t)+x_{2}(t),\\ x_{1}(0) & \;=1,\\ x_{2}(0) & \;=1, \end{array}\right.$$

(9) $$y(t)=e^{-\beta_{1}t}+e^{-\beta_{2}t}.$$

Since the model output y(t) is symmetric with respect to $\beta_{1}$ and $\beta_{2}$, interchanging the two parameters leads to identical observations. To ensure practical identifiability of individual parameters and to avoid obtaining two equivalent MLEs, we define their admissible ranges to be non-overlapping as follows:

$$\begin{array}{l} 0\leq \beta_{1}\leq 3{\text{hr}}^{-1},\\ 3\leq \beta_{2}\leq 6{\text{hr}}^{-1}. \end{array}$$

For our experiment, the true parameters are defined as $\beta_{1}=2$ hr^-1^ and $\beta_{2}=4$ hr^-1^. Observations are generated from the true model plus noise. The noise distribution is chosen to be a truncated normal distribution with mean 0 and standard deviation σ = 0.005, truncated below at $-f(x|\theta_{true})$ at each candidate location x, to ensure that the noisy observations remain non-negative.

The candidate times for measurement are from t=1 to t=10 hours in time steps of 0.1 hours. This experiment will be referred to as Experiment 1 in the following.

#### 5.1.2 Microbial growth model

While the sum of two exponentials model is simple and its ODEs are linear in parameters, we also present examples based on a nonlinear bioreactor model. This model was described by Holmberg [33], with the following mathematical form:

(10) $$\frac{d(BG)}{dt}=\frac{\mu_{\text{Max}}\times SC}{K_{S}+SC}\times BG-K_{D}\times BG,$$

(11) $$\frac{d(SC)}{dt}=-\frac{1}{{\text{Yield}}_{C}}\times \frac{\mu_{\text{Max}}\times SC}{K_{S}+SC}\times BG,$$

where $\mu_{\text{Max}}$ is the maximum growth rate, $K_{S}$ refers to the 50 % saturation constant for the substrate concentration, $K_{D}$ refers to the decay rate and ${\text{Yield}}_{C}$ is the yield coefficient. Note that the time dependence “t” is dropped from the states for the sake of notational brevity. The initial conditions used are as follows: BG(0)=1g/L and SC(0)=30g/L. The model is structurally identifiable based on the Taylor series approach for SIA given that the initial conditions are known [34].

To account for realistic biological and operational variability, we define wide parameter bounds containing the true values as follows:

$$\begin{array}{ll} \text{Maximum growth rate }(\mu_{\text{Max}}):\; & 0.1\leq \mu_{\text{Max}}\leq 50\;h^{-1},\\ \text{Saturation constant }(K_{S}):\; & 0.1\leq K_{S}\leq 50\;g/L,\\ \text{Decay constant }(K_{D}):\; & 0.0001\leq K_{D}\leq 1\;h^{-1},\\ \text{Yield coefficient }({\text{Yield}}_{C}):\; & 0.01\leq {\text{Yield}}_{C}\leq 10. \end{array}$$

To evaluate performance under different conditions, we consider the following two setups:

• **Experiment 2** has a single output where only *bacterial growth (BG)* is observed. The only unknown parameters are $\mu_{\text{Max}}$, $K_{D}$, and ${\text{Yield}}_{C}$, while $K_{S}$ is fixed at 30g/L (see below for details).
• **Experiment 3** has two outputs where both *bacterial growth (BG)* and *substrate concentration (SC)* are observed. In this case, all model parameters are treated as unknown.

The true function that simulates bacterial growth and substrate concentrations is based on parameter values adapted from [33]. All of the algorithms applied are tasked with establishing practical identifiability for the model parameters. The parameters in each experiment and their true values are given as follows:

• **Experiment 2:** $\mu_{\text{Max}}=1\;h^{-1}$, $K_{D}=0.1\;h^{-1}$, and ${\text{Yield}}_{C}=0.5$
• **Experiment 3:** $\mu_{\text{Max}}=0.5\;h^{-1}$, $K_{S}=30\;g/L$, $K_{D}=0.05\;h^{-1}$, and ${\text{Yield}}_{C}=0.6$.

These parameter values fall within biologically plausible ranges and are chosen to ensure that no negative values are generated within the simulation time frame. The decision to fix $K_{S}$ was based on a practical identifiability analysis conducted for Experiment 2 using the MATLAB tool Data2Dynamics [26], which determined that either $\mu_{\text{Max}}$ or $K_{S}$ had to be fixed in order for Experiment 2 to be practically identifiable. The choice to fix $K_{S}$ was further justified by its low sensitivity within the model (see Appendix B). The candidate times for measuring in both experiments are from t=1 to t=60 hours, with time steps of 0.5 hours. All time ranges were selected to capture the system’s full dynamic behaviour, while the step sizes ensure a sufficiently smooth resolution of the dynamics. These settings can be freely adjusted to suit specific experimental designs.

The standard deviation for the noise is based on the typical ranges of the observed output. For Experiment 2, which only has bacterial growth as the output, the standard deviation $\sigma_{BG}$ of the noise distribution is set to 2. For Experiment 3, $\sigma_{BG}$ is set to 0.5 and $\sigma_{SC}$ to 1.6. A larger noise level is used in Experiment 2 to increase the difficulty of establishing practical identifiability, which allows us to better distinguish algorithm performance. The noise distribution is again chosen to be a truncated normal distribution.

A sensitivity analysis was performed to evaluate how model output variances respond to changes in parameters. Results for Experiments 1, 2, and 3 are presented in Appendix B.

### 5.2 Speed of determining practical identifiability

**Fig. 2:**
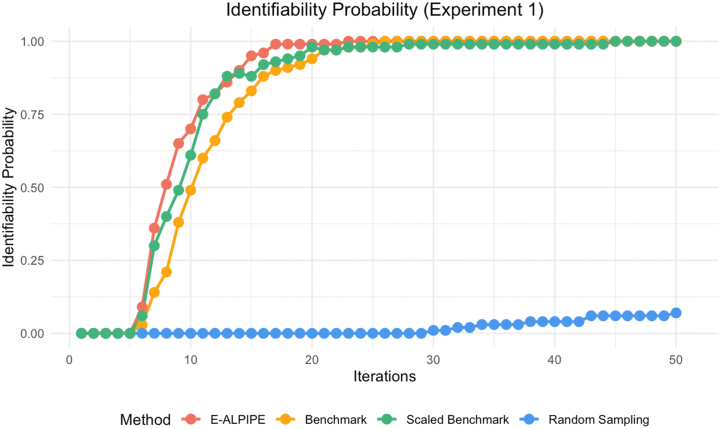
Practical identifiability probability over iterations for Experiment 1.

**Fig. 3:**
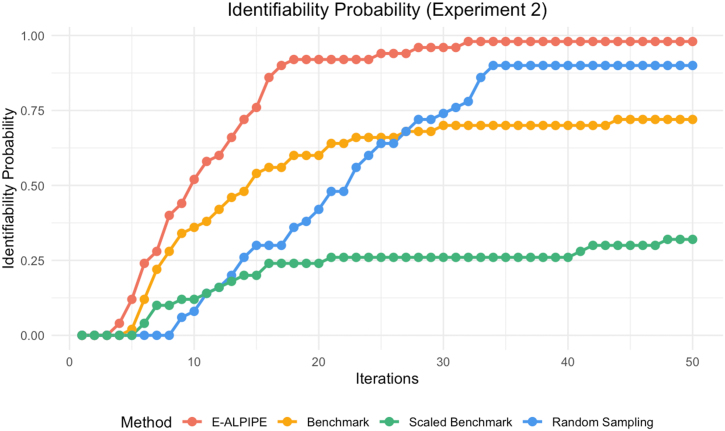
Practical identifiability probability over iterations for Experiment 2.

**Fig. 4:**
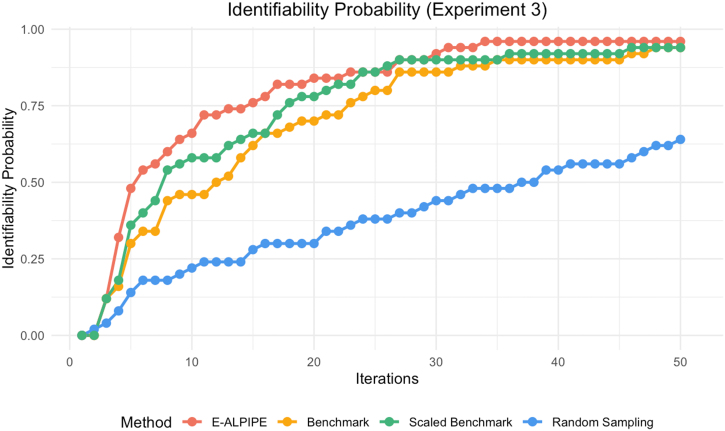
Practical identifiability probability over iterations for Experiment 3.

Based on the results shown in Fig. 2, Fig. 3, Fig. 4, the E-ALPIPE algorithm exhibits a significantly faster increase in identifiability probability across iterations compared to the other methods. Initially, E-ALPIPE achieves a steeper ascent in all experiments, suggesting that it is more efficient in achieving practical identifiability results with fewer iterations. This demonstrates that our algorithm is more effective in selecting data points that provide significant information on model’s practical identifiability.

**Fig. C.13:**
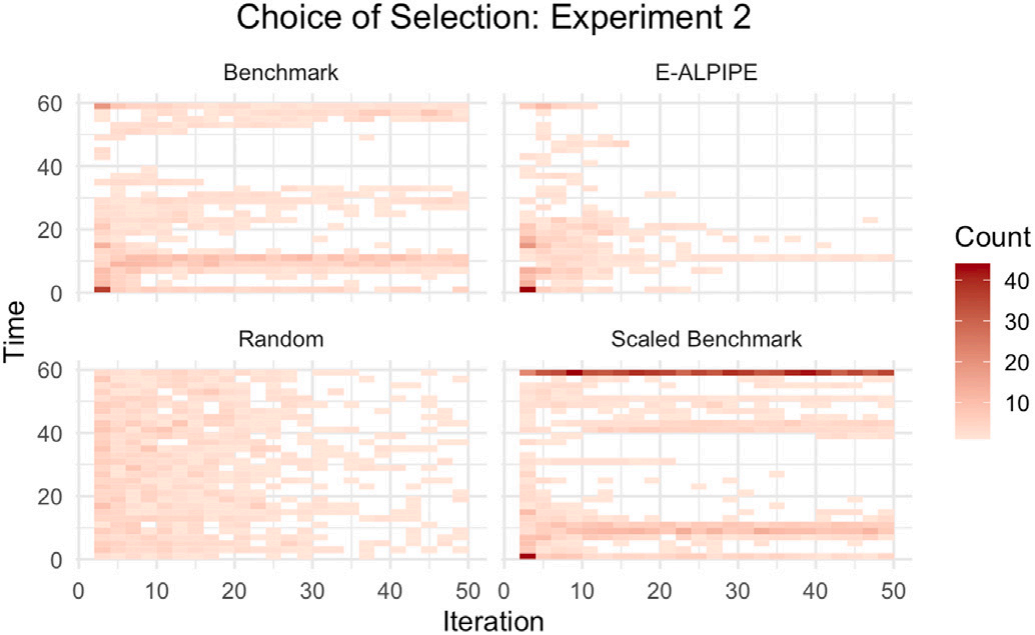
Heatmap of sampling locations selected by each algorithm in Experiment 2.

We note that in Fig. 3, the benchmark and scaled benchmark algorithms perform worse than the random baseline. This unexpected result may be due to the benchmark repeatedly selecting locations with high variance, leading to oversampling in those regions. Especially the scaled benchmark, which further divides by the estimated noise, may underestimate the information in some noisy areas. As shown in Fig. C.13, the oversampling of the scaled benchmark is apparent. In contrast, while E-ALPIPE also focuses on informative areas, it demonstrates more exploratory behaviour. The selected sampling locations for each method are visualised as heatmaps in Appendix C.

In Fig. 4, the scaled benchmark outperforms the original benchmark. This highlights the benefit of normalising the disagreement criterion by the estimated noise variance, which helps prevent the selection from being biased toward outputs with inherently higher noise. Without this adjustment, the benchmark may be misled by outputs that exhibit large variability but also have a lot of noise, providing limited information for identifiability.

We also report the average number of iterations required to establish practical identifiability in Table 1. For replications that failed to achieve practical identifiability, we assign a value equal to the maximum number of iterations when computing the average. We highlight the method with the smallest average number of iterations required to achieve practical identifiability. Compared to the second-best algorithm, E-ALPIPE requires a significantly smaller amount of data according to the Wilcoxon signed-rank test. On average, it reduces the number of samples required to confirm practical identifiability by 6.4 %, 48 %, and 18 % for Experiment 1, 2 and 3, respectively.

**Table 1:** Average number of iterations required to confirm practical identifiability across methods and experiments. An asterisk (*) is used to denote methods that are statistically significantly better than all others.

Method	Experiment 1	Experiment 2	Experiment 3
E-ALPIPE	$9.46^{*}$	$12.14^{*}$	$11.4^{*}$
Benchmark	11.7	23.38	16.16
Scaled Benchmark	10.11	39.52	13.94
Random	49.2	23.98	38.28

### 5.3 Width of the profile likelihood confidence interval

**Fig. 5:**
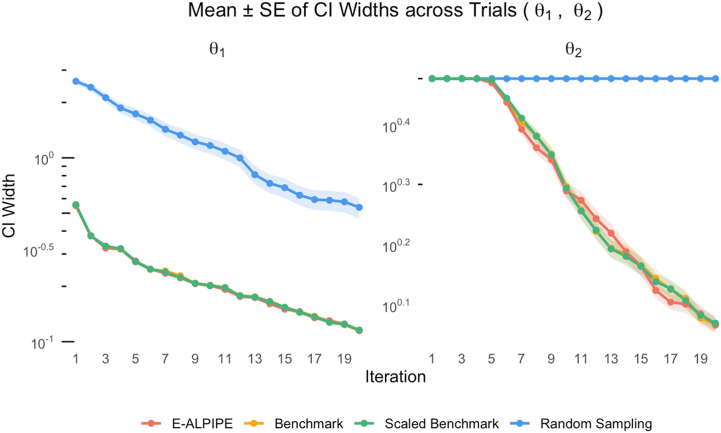
Profile-likelihood CI width as a function of the number of iterations for Experiment 1. Each line represents the mean CI width (± SE) across replicates.

**Fig. 6:**
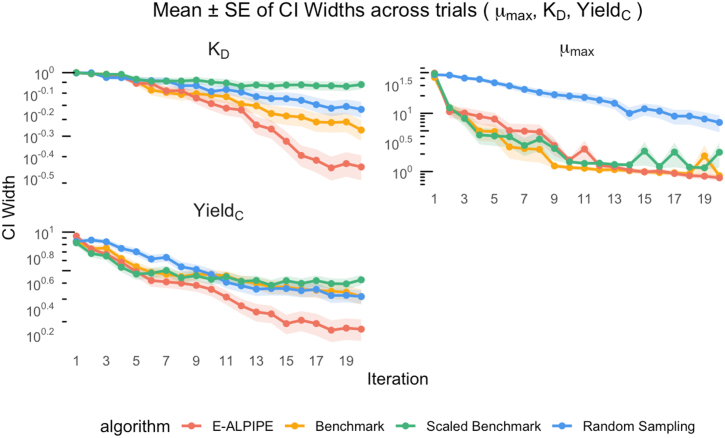
Profile-likelihood CI width as a function of the number of iterations for Experiment 2. Each line represents the mean CI width (± SE) across replicates.

**Fig. 7:**
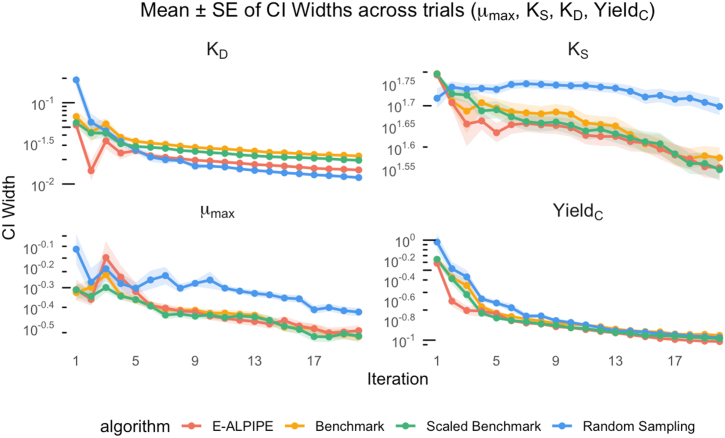
Profile-likelihood CI width as a function of the number of iterations for Experiment 3. Each line represents the mean CI width (± SE) across replicates.

To evaluate each algorithm’s ability to produce precise parameter estimates, we first visualise the trajectories of the logarithm of CI widths (Fig. 5, Fig. 6, Fig. 7) over the first 20 iterations. The CI width serves as a proxy for estimator precision; narrower intervals suggest greater precision of the estimated parameter values. As shown in the figures, E-ALPIPE either achieves or closely approaches the smallest CI widths across iterations in all experiments. For statistical inference, we compare the CI widths after each algorithm has collected exactly 20 observations using the Wilcoxon signed-rank test. Note that, for a fair comparison across methods, we fix the number of observations to 20, and do not terminate the algorithm early even if practical identifiability is achieved before reaching 20 observations, so that each algorithm is evaluated using the same amount of data. If a sampling strategy fails to achieve practical identifiability within 20 observations, we assign the full parameter search interval (as defined in Section 5.1) as its CI width for the purpose of averaging. Summary statistics of the resulting CI widths and their associated standard errors are presented in Table 2, Table 3, Table 4. In each column, the value with the smallest mean is highlighted. The results show that E-ALPIPE performs best in most cases, with no statistically significant differences among E-ALPIPE, Benchmark, and Scaled Benchmark in general. The isolated case where Random sampling performs best (Experiment 3, parameter $K_{D}$) is not sufficient to demonstrate its overall effectiveness, as it performs significantly worse than the other methods in estimating the other parameters, and also in most other settings. These results demonstrate that E‑ALPIPE consistently achieves the narrowest or near-narrowest profile-likelihood intervals across most of the parameters and experiments, indicating higher or at least comparable estimator precision under the same data budget.

**Table 2:** Experiment 1: Mean width (± standard error) of the CIs for parameters $\theta_{1}$ and $\theta_{2}$.

Method	$\text{CI}(\theta_{1})\;({\text{hr}}^{-1})$	$\text{CI}(\theta_{2})\;({\text{hr}}^{-1})$
E-ALPIPE	$0.115\pm 3.05\times 10^{-3}$	1.16±0.0341
Benchmark	$0.115\pm 3.68\times 10^{-3}$	1.17±0.0331
Scaled Benchmark	$0.116\pm 2.75\times 10^{-3}$	1.18±0.0334
Random	0.538±0.0689	3.00±0.00

**Table 3:** Experiment 2: Mean width (± standard error) of the CIs for the parameters $\mu_{\text{Max}}$, $K_{D}$ and $Yield_{C}$.

Method	$\text{CI}(\mu_{\text{Max}})\;({\text{hr}}^{-1})$	$\text{CI}(K_{D})\;({\text{hr}}^{-1})$	$\text{CI}(Yield_{C})$
E-ALPIPE	0.780±0.0819	0.357±0.0488	1.75±0.333
Benchmark	0.848±0.0883	0.535±0.0604	3.14±0.436
Scaled Benchmark	2.14±0.978	0.878±0.0434	4.24±0.389
Random	6.97±2.27	0.670±0.0580	3.14±0.380

**Table 4:** Experiment 3: Mean width (± standard error) of the CIs for the parameters $\mu_{\text{Max}}$, $K_{S}$, $K_{D}$, and $Yield_{C}$.

Method	$\text{CI}(\mu_{\text{Max}})\;({\text{hr}}^{-1})$	$\text{CI}(K_{S})\;(g/L)$	$\text{CI}(K_{D})\;({\text{hr}}^{-1})$	$\text{CI}(Yield_{C})$
E-ALPIPE	0.334±0.0185	38.9±2.27	$0.0151\pm 3.32\times 10^{-4}$	$0.0973^{*}\pm 1.52\times 10^{-3}$
Benchmark	0.311±0.0173	40.1±2.23	$0.0224\pm 5.53\times 10^{-4}$	$0.112\pm 2.09\times 10^{-3}$
Scaled Benchmark	0.317±0.0184	38.6±2.26	$0.0199\pm 5.50\times 10^{-4}$	$0.104\pm 1.98\times 10^{-3}$
Random	0.398±0.0156	52.1±1.90	$0.0121^{*}\pm 2.44\times 10^{-4}$	$0.108\pm 2.63\times 10^{-3}$

### 5.4 Assessing the point estimate quality

Another way to assess the quality of our sampling algorithms is to evaluate whether the data acquired by each sampling strategy yield parameter estimates that are capable of reproducing the true system dynamics. The predicted function value at each iteration is obtained by substituting the MLEs into the given model. To reduce the risk of convergence to local optima, we apply a multi-start optimisation approach when estimating the MLEs.

To compute the difference between the predicted and true functions, we look at the output over a sequence of time points T, corresponding to the candidate time points specified in Section 5.1. At each time point t∈T, we calculate the absolute difference between the predicted value $f(t,\hat{\theta })$ and the true value $f(t,\theta_{\text{true}})$, and then average these differences over all time points:

(12) $$Diff=\frac{1}{T}\sum_{t}\left|f(t,\hat{\theta })-f(t,\theta_{\text{true}})\right|.$$

This metric represents the mean absolute deviation between the predicted and true values across all time points in the candidate set.

As in the previous section, we visualise the mean prediction error across iterations and perform Wilcoxon signed-rank tests on the prediction errors after each algorithm has collected exactly 20 observations. Each algorithm was terminated after exactly 20 observations to ensure a fair and consistent comparison across methods.

**Fig. 8:**
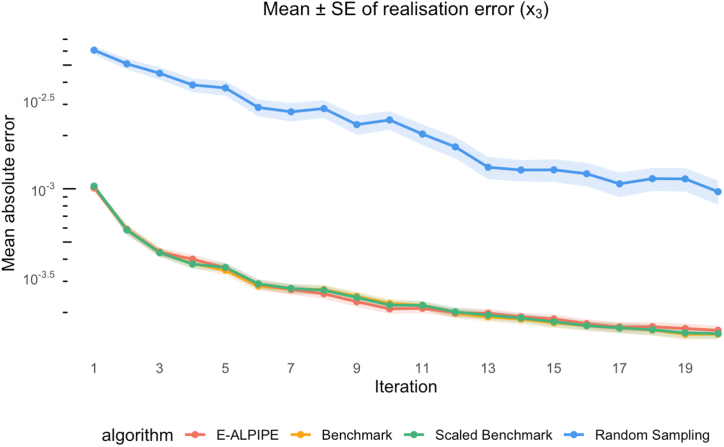
Mean absolute difference (± SE) between predicted and true observable y(t) as a function of iteration for Experiment 1.

**Fig. 9:**
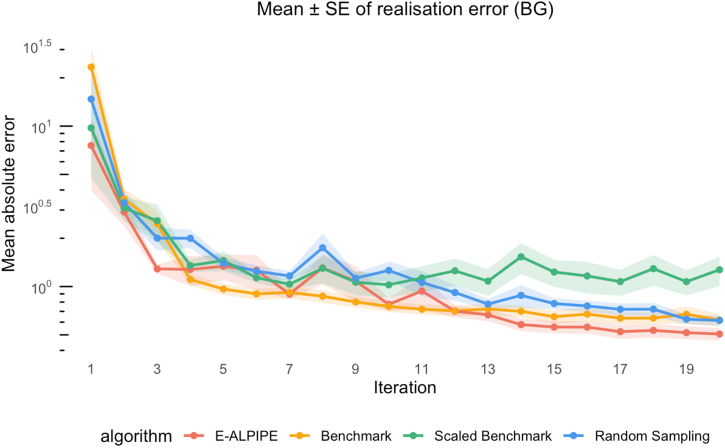
Mean absolute difference (± SE) between predicted and true observable y(t) as a function of iteration for Experiment 2.

**Fig. 10:**
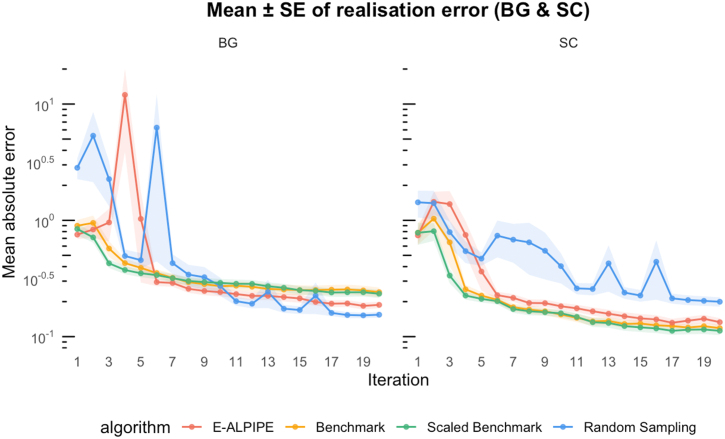
Mean absolute difference (± SE) between predicted and true observable y(t) as a function of iteration for Experiment 3.

From the plots (Fig. 8, Fig. 9, Fig. 10), we observe that E-ALPIPE consistently performs best in most cases, with the exception of the BG and SC outputs of Experiment 3. In that experiment, Random sampling achieves the best performance, while Benchmark and Scaled Benchmark perform worst for the BG output, with the reverse pattern observed for the SC output. E-ALPIPE provides a balanced MLE performance in this case. Even in scenarios where it is not the best-performing method, the difference from the best is small, indicating robust and consistently high performance across experiments. Table 5, Table 6, Table 7 report the mean and standard error of this metric for each observable and sampling strategy across the three experiments, based on 20 collected observations per replication. The method which has the smallest average absolute difference is highlighted. Although the Wilcoxon signed-rank test indicates that the performance difference between the best and second-best approach is often not statistically significant, E-ALPIPE consistently achieves the lowest or near-lowest average absolute differences in nearly all cases. These results suggest that E-ALPIPE provides more accurate model dynamics under a fixed data budget in most practical scenarios.

**Table 5:** Experiment 1: Averaged absolute difference (± standard error) between predicted and true observable y(t).

Method	${\text{Diff}}_{y}$
E-ALPIPE	$1.59\times 10^{-4}\pm 9.62\times 10^{-6}$
Benchmark	$1.51\times 10^{-4}\pm 9.66\times 10^{-6}$
Scaled Benchmark	$1.52\times 10^{-4}\pm 9.79\times 10^{-6}$
Random	$9.62\times 10^{-4}\pm 1.50\times 10^{-3}$

**Table 6:** Experiment 2: Average absolute difference (± standard error) between predicted and true output for BG.

Method	${\text{Diff}}_{BG}$
E-ALPIPE	0.506±0.0464
Benchmark	0.619±0.0553
Scaled Benchmark	1.27±0.266
Random	0.614±0.0445

**Table 7:** Experiment 3: Averaged absolute difference (± standard error) between predicted and true outputs BG and SC.

Method	${\text{Diff}}_{\text{BG}}$	${\text{Diff}}_{\text{SC}}$
E-ALPIPE	0.187±0.0120	0.133±0.0108
Benchmark	0.241±0.0231	$0.118\pm 8.01\times 10^{-3}$
Scaled Benchmark	0.234±0.0186	$0.112\pm 9.40\times 10^{-3}$
Random	$0.154\pm 8.60\times 10^{-3}$	0.200±0.0183

## 6 Discussion

The experimental results demonstrate the effectiveness of the E-ALPIPE algorithm across three criteria relevant to practical identifiability analysis. In particular, E-ALPIPE outperforms the benchmark algorithm and random sampling method in terms of the speed with which practical identifiability is achieved, the precision of parameter estimates as measured by confidence interval widths, and the accuracy of system dynamics reproduction.

To further understand how the algorithms prioritise sampling locations, we conducted a sensitivity analysis and provided heat maps of the chosen observations over time (see Appendix B, Appendix C). The Benchmark and Scaled Benchmark algorithms tend to focus on regions of high local sensitivity, potentially leading to over-sampling of narrow informative regions. In contrast, E-ALPIPE demonstrates a more exploratory behaviour, distributing observations more broadly across the design space, which may contribute to its robustness and overall performance across different scenarios.

Since E-ALPIPE evaluates the profile likelihood only at the boundaries—rather than over the full parameter space—it also achieves substantial reductions in computational cost. This efficiency makes it especially suitable for computationally expensive models, where full-profile evaluations become prohibitive. In particular, E-ALPIPE evaluates only 2N+1 boundary-based models per iteration, compared to approximately 10N models in the Benchmark and Scaled Benchmark approaches, leading to a markedly smaller number of optimisations that need to be performed. In our implementation, E-ALPIPE required approximately 55 % less computation time to determine the next experiment than the Scaled Benchmark and Benchmark algorithms. For example, in Experiment 3, it required only 14 s per iteration compared to 31 s for Benchmark on average. When combined with the smaller number of iterations required to reach practical identifiability (Table 1), the total runtime reduction typically ranged between 60 % and 75 % across experiments.

**Fig. B.11:**
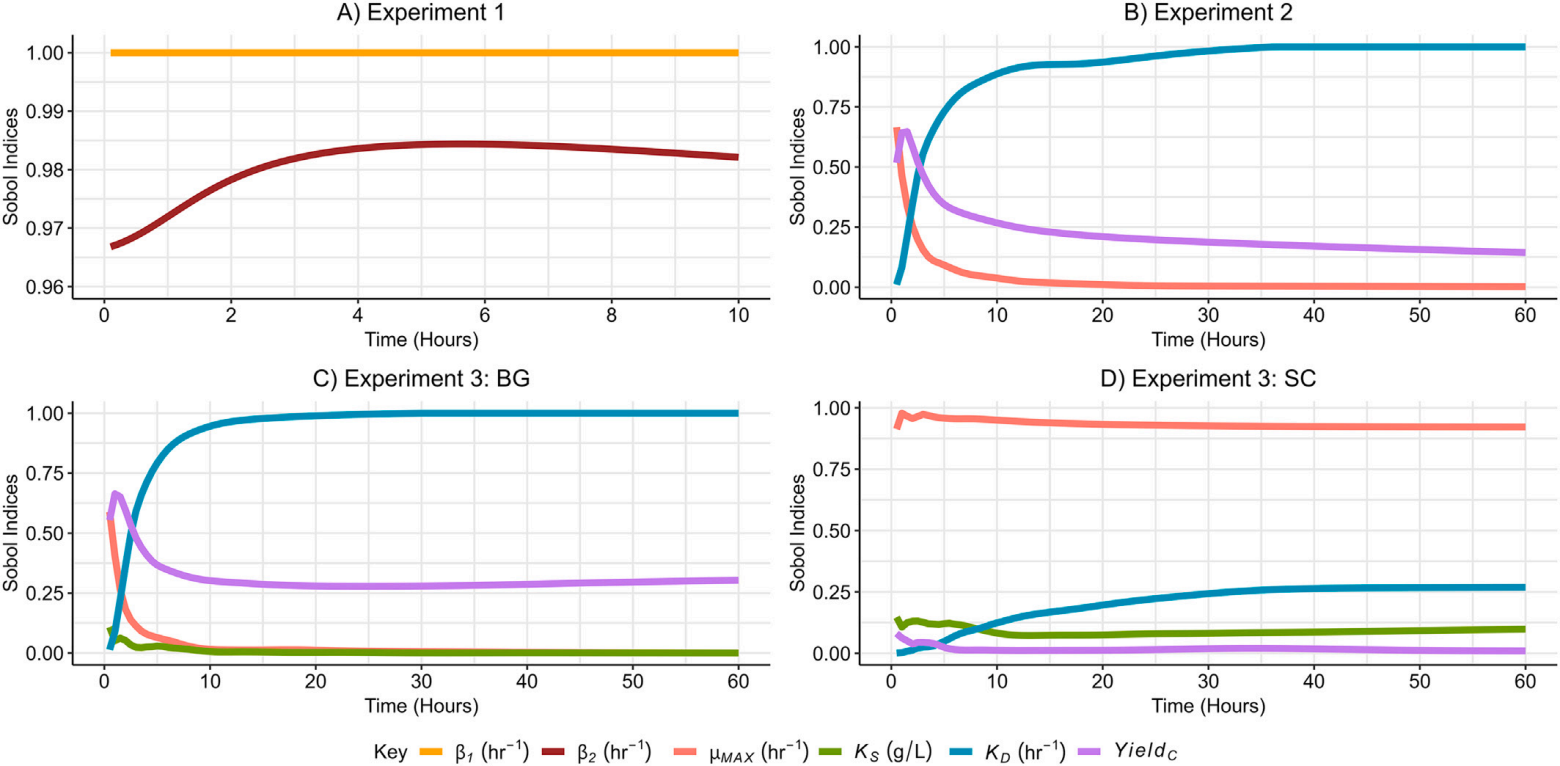
Sensitivity analyses showcasing the Sobol indices for Experiment 1 (A), Experiment 2 (B) and Experiment 3 (C and D) based on the model equations, experiment settings and parameter lower and upper bounds for each experiment (see Section 5.1).

**Fig. C.14:**
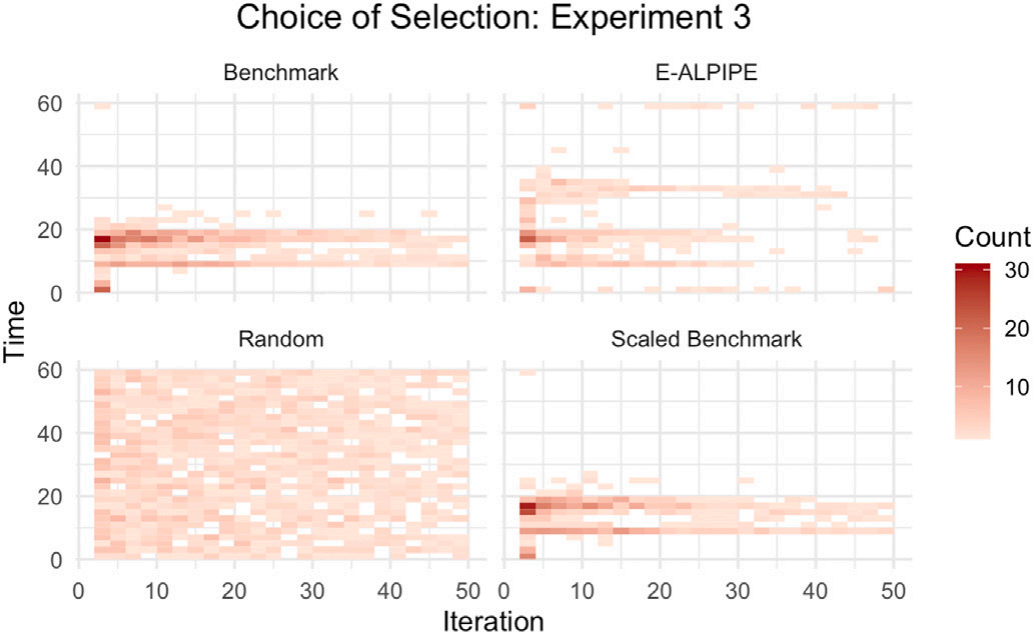
Heatmap of sampling locations selected by each algorithm in Experiment 3.

E-ALPIPE also successfully detects practical identifiability even for parameters with intrinsically low sensitivity, such as $K_{D}$ in Experiment 3 (see Appendix B, Fig. B.11). Based on Fig. C.14 in Appendix C, the Benchmark, E-ALPIPE and Scaled Benchmark primarily focused on sampling locations within the first 20 hours of the experiment. However, the E-ALPIPE also briefly explored sampling locations between 25 and 40 hours which correspond to higher sensitivity values for the poorly identifiable parameter $K_{D}$ in Fig. B.11 (Experiment 3). The algorithms that did not explore this space, on average achieved practical identifiability at a later stage compared to the E-ALPIPE. This further confirms that E-ALPIPE will aim to target informative sampling locations in order to practically identify parameters.

While in the present study, E-ALPIPE has been applied to a sum of exponentials model and microbial growth models, E-ALPIPE can be applied to both linear and non-linear ODEs which are used as the basis for many practically relevant models including biological and clinical models. Currently, the code is available to use in R^1^ and simply requires the user to input the structurally identifiable model, assumed noise distribution and parameter limits. E-ALPIPE will return the recommended next sampling location based on these inputs.

### Limitations

The present bisection search for profile-likelihood CIs assumes unimodal profiles and may yield overly wide intervals or fail to detect practical identifiability under strong multimodality. If it is expected that profile likelihoods are highly multimodal, one can instead employ grid/multi-interval search to ensure that threshold crossings are not missed. Note that this would increase the computational cost but not impact experimental efficiency of E-ALPIPE. All evaluations to date were conducted on synthetic data; validation with real biological measurements is still required. Finally, our experiments involved at most four unknown parameters and two observables; scalability to higher-dimensional models has not yet been systematically assessed.

### Future work

We plan to extend E-ALPIPE beyond the unimodal setting by developing procedures that explicitly detect and efficiently handle multimodal likelihoods (e.g., multi-interval detection). Meanwhile, we would like to explore design strategies for continuous-time protocols and time-series acquisitions rather than single time-point measurements. A further line of work is scalability: although our present study involves up to four unknown parameters and two observables, many bioengineering models feature substantially larger parameter spaces and multiple readouts. We will investigate computational strategies such as parallelisation to reduce the cost of repeated PIA checks in higher dimensions.

We also aim to validate the method on real biological systems. As part of our future work, we will test the algorithm on pharmacokinetic/pharmacodynamic models which are used to estimate important clinically relevant parameters. At present, many clinical trials collect repeated measurements which can be invasive (e.g., plasma sampling). In moving from synthetic to real datasets, we anticipate additional challenges, including measurement limits and correlated errors across time or observables. Within our framework, these can be accommodated by using appropriate likelihood formulations (e.g. truncated models) and by developing a covariance-aware metric. These models can also be used with E-ALPIPE to determine the best experimental setup and reduce the number of measurements that are needed from each patient. Finally, to facilitate adoption and community testing on more complicated models, we plan to release a user-friendly software interface built on our public implementation.

## 7 Conclusion

Our results demonstrate that the E-ALPIPE algorithm is an effective and resource-efficient strategy for active data collection in practical identifiability analysis. Across three synthetic case studies, the algorithm consistently achieved practical identifiability more quickly, produced narrower profile-likelihood confidence intervals, and reproduced system dynamics more accurately than a state-of-the-art benchmark and random sampling. These advantages arise from E-ALPIPE’s greedy information-gain criterion, which targets practical identifiability by directly maximising the disagreement between the current predictive model and a constrained optimised model evaluated at the parameter boundaries. Our algorithm maintained its performance across models that differ in structure and dimensionality, highlighting its generalisability.

For experimentalists, these gains translate directly into reduced laboratory costs and time. Practical identifiability sets a lower bound on the data collection required for trustworthy parameter estimation; accelerating its confirmation therefore increases the robustness of model-based study design in bioengineering.

In summary, E-ALPIPE combines computational tractability with strong statistical performance, offering a practical path toward data-efficient experimental design in computational biology.

## CRediT authorship contribution statement

**Xiaolu Liu:** Conceptualization, Methodology, Visualization, Writing – original draft, Writing – review & editing. **Linda Wanika:** Conceptualization, Methodology, Visualization, Writing – original draft, Writing – review & editing. **Michael J. Chappell:** Supervision, Writing – review & editing. **Juergen Branke:** Conceptualization, Methodology, Supervision, Writing – review & editing.

## Declaration of competing interest

The authors declare that they have no known competing financial interests or personal relationships that could have appeared to influence the work reported in this paper.

## Figures and Tables

**Fig. C.12:**
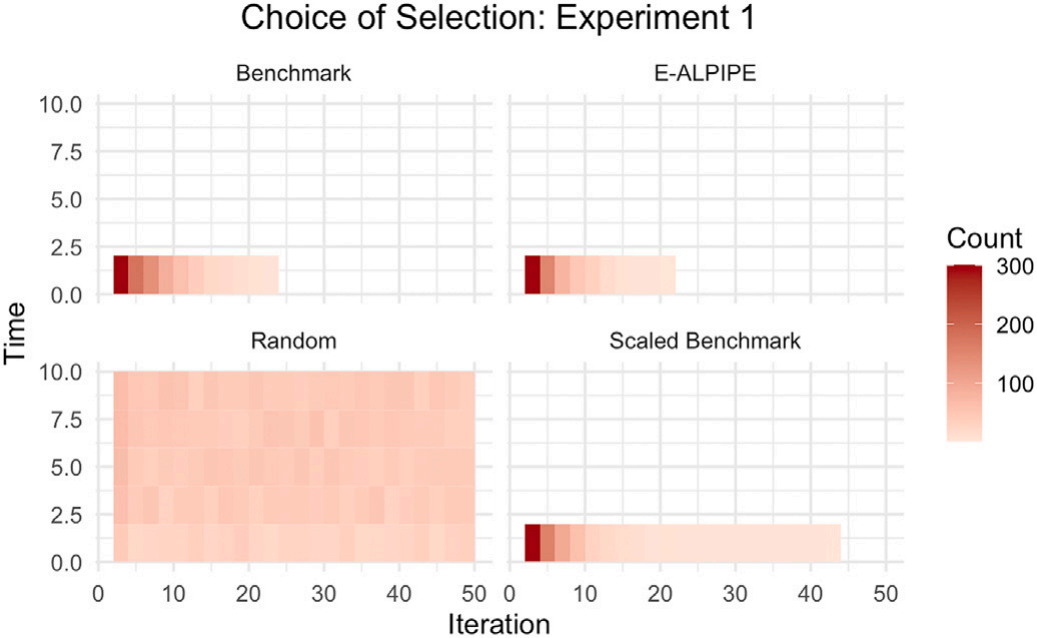
Heatmap of sampling locations selected by each algorithm in Experiment 1.

## Data Availability

All code used for implementing the algorithm, conducting experiments, and performing data analysis is available in a GitHub repository at https://github.com/lulu0120/E-ALPIPE-Algorithm. We have also used Zenodo to assign a DOI to the repository: 10.5281/zenodo.16411995

## Appendix A Calculation of noise variance under truncated normal assumption

To account for the physical constraint that measurements cannot be negative, we model the measurement noise using a normal distribution truncated below at zero. Based on the likelihood definition in Eq. (3), the truncated likelihood is expressed as

(A.1) $$L(\theta |D,\sigma )=\prod_{j=1}^{n}\frac{\frac{1}{\sigma }\;\phi \;\left(\frac{y_{j}-f(x_{j}|\theta )}{\sigma }\right)}{1-\Phi \;\left(\frac{-f(x_{j}|\theta )}{\sigma }\right)},$$

where Φ denotes the standard normal CDF.

At each location x, we assume the measurement Y follows a truncated normal distribution, $Y∼N(\mu ,\sigma^{2})$ while restricting Y≥0. Letting ℓ=0 be the lower limit and $\alpha =\frac{\ell -\mu }{\sigma }$, the density is proportional to the original normal PDF for values Y≥ℓ. Suppose μ is the predicted value from the MLE at that location and σ is a given standard deviation, then the estimated variance of Y is given by

(A.2) $$\text{Var}[Y|Y\geq \ell ]=\sigma^{2}\left[1-\frac{\alpha \;\phi (\alpha )}{1-\Phi (\alpha )}-{\left(\frac{\phi (\alpha )}{1-\Phi (\alpha )}\right)}^{2}\right],$$

where $\phi (\alpha )=\frac{1}{\sqrt{2\pi }}e^{-\alpha^{2}/2}$, and $\;\Phi (\alpha )=\int_{-\infty }^{\alpha }\frac{1}{\sqrt{2\pi }}e^{-z^{2}/2}\;dz$ denote the standard normal probability density and cumulative distribution functions, respectively.

We estimate the noise variance at each sampling location as follows:

1. Evaluate the model prediction $\mu =f(x|\hat{\theta })$, where $\hat{\theta }$ is the current MLE.
2. Compute α=(ℓ−μ)/σ.
3. Evaluate ϕ(α) and Φ(α).
4. Substitute into Eq. (A.2) to compute the variance of the truncated normal distribution.

This noise variance estimate is used to normalise model uncertainty in both the E-ALPIPE and Scaled Benchmark algorithms, where the estimated noise level informs the sampling selection stage.

## Appendix B Sensitivity analysis

A sensitivity analysis was performed in R based on the Sobol Indices approach [35]. In short, the total variance of a model’s output is separated and attributed to each of the model parameters and inputs of the model system. Sobol Indices provide a quantitative way of analysing the sensitivity of the model outputs based on the model parameters, where higher values indicate that the variance of the model’s outputs is sensitive to these parameters at a particular location, and low values indicate that the variance of the model’s output is less sensitive to these specific parameters.

For Experiment 1 (Fig. B.11A) the Sobol indices for both $\theta_{1}$ and $\theta_{2}$ are above 0.9 throughout the entire experiment duration. For the bacterial growth observations in Experiments 2 and 3: For the BG output (Fig. B.11B and C), the parameter $K_{D}$ has the highest Sobol indices which are above 0.9. The parameter $Yield_{C}$, has Sobol indices above 0.5 during the first five hours of the experiment duration but then these decrease to less than 0.3 afterwards. The parameter $K_{S}$ has the lowest Sobol indices (<0.2) overall for both the bacterial growth observation and the substrate concentration (Fig. B.11D). The parameter $\mu_{\text{MAX}}$ has a higher Sobol index for the substrate concentration (>0.9) output compared to the bacterial growth output.

## Appendix C Choice of sampling locations

To illustrate the measurement location selection across different sampling methods, Fig. C.12, Fig. C.13, Fig. C.14 present heatmaps of selected locations aggregated over all independent replications, prior to achieving practical identifiability for all parameters. The plots demonstrate that in Experiments 2 and 3, E-ALPIPE, Benchmark, and Scaled Benchmark tend to select locations corresponding to regions of high Sobol sensitivity indices (Fig. B.11), confirming that these algorithms prioritise informative regions in the design space.

For Experiment 1, however, the sensitivity analysis suggests that regions beyond t=4 hours provide limited information for $\theta_{2}$ due to the dominance of noise relative to the variability introduced by $\theta_{2}$. Both the Benchmark and E-ALPIPE algorithms correctly focus on earlier time points (before t=2.5), effectively accounting for the noise level in their selection. Additionally, in Fig. C.13, the Benchmark algorithm exhibits more frequent repeated selections at certain locations, indicating potential over-exploitation of specific regions, whereas E-ALPIPE explores a broader set of informative regions. Note that E-ALPIPE and the Benchmark algorithm often achieve practical identifiability before iteration 50; hence the heatmaps are lighter in later iterations or stop entirely.

## Footnotes

1 GitHub repository: https://github.com/lulu0120/E-ALPIPE-Algorithm
